# Effect of Sijunzi Pills on Pharmacokinetics of Omeprazole in Beagle Dogs by HPLC-UV: A Herb-Drug Interaction Study

**DOI:** 10.1155/2021/4181196

**Published:** 2021-11-26

**Authors:** Hua-ling Xia, Xin-jie Zhao, Yu-ji Zhang, Xiao-hang Su, Bo Sun, Xiang-jun Qiu

**Affiliations:** ^1^Luoyang Orthopedic-Traumatological Hospital of Henan Province, Henan Provincial Orthopedic Hospital, Luoyang 471003, China; ^2^School of Basic Medical Sciences, Henan University of Science and Technology, Luoyang 471023, China

## Abstract

A sensitive high-performance liquid chromatography (HPLC-UV) method for determination of omeprazole in beagle dog plasma was developed and to investigate the effect of Sijunzi pills (SJZPs) on the pharmacokinetics of omeprazole in beagle dogs. The beagle dog plasma was extracted with ethyl acetate and n-hexane under alkaline conditions. Omeprazole and internal standard (IS, fluconazole) were separated on an XDB-C18 column, and acetonitrile and 0.1% trifluoroacetic acid were used as the mobile phase. Omeprazole and IS were detected by using a diode array detector. This experiment adopts the experimental design of double-cycle self-control. In the first cycle (group A), six beagle dogs were given omeprazole 0.67 mg/kg orally in a single dose. In the second period (group B), the same six beagle dogs were orally given SJZPs 0.2 g/kg twice a day for 7 consecutive days, and then, omeprazole was orally given. At the different time points after omeprazole was given in the two periods, the blood samples were collected. The concentration of omeprazole was detected by the developed HPLC method. DAS 2.0 was used to calculate the pharmacokinetic parameters of omeprazole. Under the current experimental conditions, this UPLC method showed good linearity in the detection of omeprazole. Interday and intraday precision did not exceed 10%, and the range of accuracy values were from −1.43% to 2.76%. The results of extraction recovery and stability met the requirements of FDA approval guidelines of bioanalytical method validation. The *C*_max_ of omeprazole in group B was 61.55% higher than that in group A, and the AUC_(0−*t*)_ and AUC_(0−∞)_ of omeprazole in group B were 63.96% and 63.65% higher those that in group A, respectively. At the same time, the clearance (CL) and apparent volume of distribution (Vd) decreased in group B. In this study, an HPLC method for the determination of plasma omeprazole concentration was established. SJZPs could inhibit the metabolism of omeprazole and increase the concentration of omeprazole in beagle dogs. It is suggested that when SJZPs are combined with omeprazole, attention should be paid to the herb-drug interactions and possible adverse reactions.

## 1. Introduction

Sijunzi Decoction (SJZD), derived from the prescription of *Taiping Huimin Heji Bureau,* is a classic representative prescription of Traditional Chinese Medicine (TCM) for supplementing *qi*. SJZD is composed of *Codonopsis pilosula*, *Atractylodes macrocephala*, *Poria cocos*, and roasted licorice. In the prescription, *Codonopsis pilosula* is the king's medicine, *Atractylodes macrocephala* is the minister's medicine, *Poria cocos* is the adjuvant, licorice is the enabling drug, and the four drugs are combined to supplement the spleen and *qi* [1]. Sijunzi pill (SJZP) is a patent medicine made of SJZD. The preparation method is as follows: 200 g *Codonopsis pilosula*, 200 g *Atractylodes macrocephala* (fried), 200 g *Poria cocos*, and 100 g roasted licorice were crushed into fine powder, sifted, and mixed evenly. The pills were panned with the decoction (50 g *ginger* and 100 g *jujube*, decocted and filtered with water several times) and dried. It has the function of supplementing *qi* and strengthening the spleen [2].

SJZD can significantly improve the treatment effect, effectively reduce the occurrence of adverse reactions, and improve the quality of life after treatment [3]. SJZD is effective and safe in patients with gastric ulcer of spleen stomach *qi* deficiency type with functional dyspepsia [4]. SJZD can ameliorate the local gastric inflammation and inflammations in peripheral blood leukocytes and might also reduce the incidence of stomach cancer in chronic gastritis [5]. At the same time, SJZD can have an anticancer effect. SJZD adjuvant interventional therapy has a good clinical effect on patients with primary liver cancer and can promote the recovery of liver function and immune function [6]. In the treatment of postoperative patients with gastric cancer, modified SJZD adjuvant chemotherapy can promote the early recovery of patients, reduce anxiety and depression, reduce the level of tumor markers, and inhibit tumor progression [7]. SJZD can affect NK cell activity and colon cancer proliferation, which may intervene IFN-*γ* secretion by regulating STAT3 signal and reduce the expression of PD-1/PD-L1, so as to improve NK cells and inhibit the growth of colon cancer cells [1]. In addition, SJZD has a certain therapeutic effect on heart failure after myocardial infarction in rats by regulating the imbalance of intestinal flora [8]. SJZD rescued neurons and improved neurobehavioural function in rats following cerebral ischaemia-reperfusion. The mechanism may be related to protection of the extracellular matrix followed by antiapoptotic effects [9]. SJZD can significantly improve the learning and memory, digestion, and absorption function of the brain and intestine in rats with spleen deficiency syndrome. Its effect is related to the upregulation of ghrelin protein expression in the hippocampus and small intestine [10].

Omeprazole is the first-generation proton pump inhibitor (PPI). After oral administration, omeprazole can be specially distributed in the secretory tubules of gastric mucosal parietal cells and irreversibly combined with the sulfhydryl group of H^+^ and K^+^-ATPase (also known as proton pump) in the secretory membrane of parietal cells, so as to inhibit the enzyme activity and block the final step of gastric acid secretion, Therefore, this product has a strong and lasting inhibitory effect on gastric acid secretion caused by various reasons. It was suitable for the treatment of gastric ulcer, duodenal ulcer, stress ulcer, reflux esophagitis, and gastrinoma. Omeprazole was metabolized by CYP2C19 and CYP3A4 in vivo [11] and was often used as a probe drug of CYP2C19 [12]. Omeprazole and its metabolites were time-dependent inhibitors of CYP2C19 and CYP3A4 [11]. Therefore, drug-drug interaction is often caused [11, 13].

TCM-based herbal therapies have gained increasing popularity worldwide, raising concerns of its efficacy, safety profile, and potential interactions with Western medications [14]. Clinically, SJZD and omeprazole are often used in combination to treat chronic atrophic gastritis, recurrent *Helicobacter pylori*-positive gastric ulcer, spleen stomach qi deficiency syndrome, spleen deficiency chronic gastritis, and other diseases [15–18]. SJZD was the strongest potential inhibitor of CYP2C19; therefore, clinically relevant herb-drug interactions (HDIs) should be taken once they are coadministered with a drug metabolized by CYP2C19 [19].

The detection of omeprazole has been reported; for example, the stability of enantioselectivity of omeprazole in enteric-coated preparations was determined by HPLC [20], and omeprazole in plasma was determined by HPLC [21]. In this study, a method for determination of omeprazole in beagle dog plasma by HPLC-UV was developed and performed using fluconazole as the internal standard (IS), and the effect of SJZP on the changes of pharmacokinetics of omeprazole in beagle dogs was observed.

## 2. Materials and Methods

### 2.1. Instruments

The high-performance liquid chromatograph was of Agilent 1100 series, including a G1379A online degasser, G1311A quaternionic pump, G1313A automatic sampler, G1316A column oven, G1315B diode array detector, and Agilent chemical workstation (Agilent, USA). An electronic analytical balance (AB204-A, Mettler Toledo Shanghai Instrument Co., Ltd.), vortex mixer (FZQ-2, Jiangsu Taixian medical device factory), nitrogen blowing instrument (D10, Hangzhou Lanyan Technology Co., Ltd.), etc. were used.

### 2.2. Chemicals

HPLC pure methanol and acetonitrile were purchased from Tianjin kemio Chemical Reagent Co., Ltd., trifluoroacetic acid (064k3647) was purchased from Sigma-Aldrich (Shanghai) Trading Co., Ltd., and analytical pure ethyl acetate, n-hexane, and sodium hydroxide were purchased from Tianjin No. 3 chemical reagent factory. Omeprazole (100367–201706) and fluconazole (100314–200503) were purchased from the National Institute for Food and Drug Control. Omeprazole Enteric-coated Capsules (060210301, 20 mg/capsule) were produced by CSPC Ouyi Pharmaceutical Co., Ltd., and SIZPs (3 g/bag) were produced by Hubei Guangren Pharmaceutical Co., Ltd.

### 2.3. Solutions Ready

10 mg of omeprazole reference substance was accurately weighted in a 10 mL volumetric flask and dissolved with methanol to 10 ml to obtain the omeprazole standard stock solution with a concentration of 1 mg/mL. The concentration of 100 *μ*g/mL, 10 *μ*g/mL, and 1 *μ*g/mL standard application solution was diluted to that of the standard stock solution with 0.1 mol/l sodium hydroxide (diluted temporarily). The IS stock solution was prepared in the same way and diluted with methanol to 100 *μ*g/mL internal standard application solution. All solutions were stored in a refrigerator at −4°C.

### 2.4. Chromatographic Conditions

The chromatographic column was Zorbax XDB-C18 (4.6 × 150 mm, 5 *μ*m, Agilent, USA), the protective column was the XDB-C18 protective column (4.6 × 12.5 mm, 5 *μ*m, Agilent, USA), the mobile phase was acetonitrile-0.1% TFA-water (20:10:70), the flow rate was 0.8 ml/min, the column temperature was 30°C, the wavelength gradient was used for detection, 262 nm in 1∼6 min and 302 nm in 6–8 min, and the reference wavelength was set at 380 nm.

### 2.5. Treatment of Plasma Samples

300 *μ*L the plasma sample to be tested was accurately drawn and put into a 5 mL EP tube, and 100 *μ*g/ml fluconazole IS working solution was added with 50 *μ*L. After mixing, 0.1 mol/L sodium hydroxide solution was added for 50 *μ*L. Then, 1 ml of ethyl acetate and 1 ml of n-hexane were added and mixed in vortex for 2 min. The upper organic phase was transferred into another EP tube and blown to dry in a nitrogen blowing instrument. The residue was redissolved with 100 *μ*L 20% acetonitrile-0.1 M sodium hydroxide solution, the resolution was taken into the sample bottle of the automatic sampler, and the injection volume was 20 *μ*L.

### 2.6. Method Verification

The specificity, precision and accuracy, standard curve and lower limit of quantification (LLOQ), recovery, and sample stability of the method were verified according to the guiding principles of the Bioanalytical Method Validation Guidance for Industry of the FDA and the guidelines for validation of quantitative analysis methods for biological samples in the Pharmacopoeia of the People's Republic of China.

### 2.7. Animals

Six beagles, half male and half female, weighing 6.5–7.5 kg, were purchased from Hubei Yizhicheng Biotechnology Co., Ltd., with the production license no. SCK (E) 2016–0020. The beagle dogs were raised in the experimental animal center of Henan university of science and technology. The animal experiment was approved by the animal ethics committee of Henan University of Science and Technology on March 20, 2021 (202103002). During the experiment, the operating procedures of experimental animals were strictly followed and the welfare of animals was guaranteed.

### 2.8. Experiment Design

This experiment adopts the experimental design of double-cycle self-control. In the first cycle (group A), six beagle dogs were given omeprazole 0.67 mg/kg orally in a single dose. The blank blood was collected before administration. 1.0 mL of venous blood was collected at 0.33, 0.67, 1, 1.5, 2, 2.5, 3, 4, 6, 8, and 12 hours hours after administration and centrifuged at 10000 rpm for 5 min. The plasma was separated and frozen for testing.

After a cleaning period of 1 week, the experiment of the second cycle was carried out (group B). The same six beagle dogs were orally given SJZPs 0.2 g/kg twice a day for 7 consecutive days. On the morning of the 8^th^ day, the beagle dogs were given omeprazole 0.67 mg/kg orally 30 min after SJZP administration. The blank blood was collected before administration. 1.0 mL of venous blood was collected at 0.33, 0.67, 1, 1.5, 2, 2.5, 3, 4, 6, 8, and 12 hours after administration and centrifuged at 10000 rpm for 5 min. The plasma was separated and frozen for testing.

### 2.9. Plasma Sample Detection

The plasma of beagle dogs to be tested was divided into two analytical batches. Each analytical batch includes accompanying standard curves and quality control samples.

### 2.10. Data Analysis

The plasma omeprazole concentrations in groups A and B were treated by DAS 2.0 program, and the main pharmacokinetic parameters were calculated by the statistical moment model. The differences of pharmacokinetic parameters between group A and group B were compared by the *t*-test of independent samples.

## 3. Result

### 3.1. Selectivity

**Figure 1:**
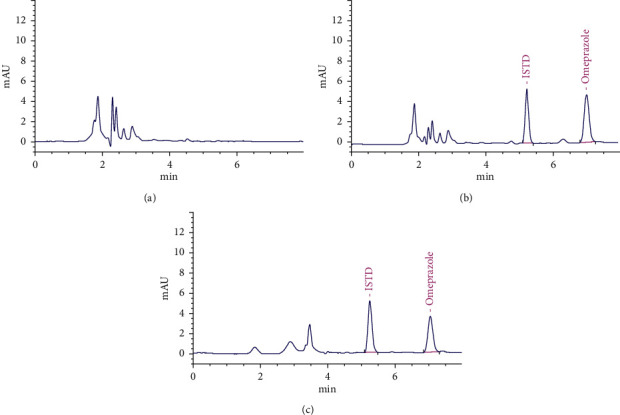
The representative chromatograms of omeprazole and IS. Blank plasma sample (a), blank plasma sample spiked with omeprazole and IS (b), and a beagle dog sample sample (c).

The chromatograms of blank beagle dog plasma, omeprazole plasma standard, and beagle dog sample after administration are shown in Figure 1. It could be seen from the figure that omeprazole and internal standard were well separated, the endogenous substances did not interfere with the detection, and the retention times of omeprazole and internal standard were 7.06 min and 5.27 min, respectively.

### 3.2. Plasma Standard Curve and Lower Limit of Quantification

The omeprazole standard working solutions with different concentrations and volumes were added successively into eight 5 mL EP tubes, and then, the blank beagle dog plasma with different volumes was added to prepare plasma samples with concentrations equivalent to 5, 10, 25, 50, 100, 250, 500, and 1000 ng/mL. After treatment according to item 2.5, the samples were tested, and the peak area of omeprazole (As) and the peak area of IS (Ai) were recorded. As/Ai was taken as the ordinate *y*, and the corresponding point concentration was taken as the abscissa *x*, and the standard curve was drawn. The regression equation of the standard curve of omeprazole was *y* = 1.5 × 10^−3^*x*–3.9 × 10^−3^ (*r* = 0.999 5). The lowest concentration of the standard curve was 5 ng/mL as the LLOQ.

### 3.3. Precision and Accuracy

The low, medium, and high concentrations (10, 250, and 750 ng/ml) of omeprazole plasma standard solution were prepared with 6 copies of each concentration and then were treated according to the plasma sample treatment method in item 2.5. Each concentration was measured in the same day, and the intraday precision was calculated. The same measurement was carried out for 3 consecutive days to calculate the interday precision. The precision was expressed by relative standard deviation (RSD %), and the accuracy was expressed by relative error (RE %). The results are shown in Table 1. The precision (% RSD) did not exceed 7.46%, and the accuracy (% RE) was in the range from −0.83% to 1.63% at low, medium, and high concentrations and met the requirements of validation.

### 3.4. Absolute Recovery

The low, medium, and high concentrations (10, 250, and 750 ng/ml) of omeprazole plasma standard solution were prepared with 6 copies of each concentration and then were treated according to the plasma sample treatment method in item 2.5, the samples were tested, and the peak area of omeprazole was recorded as A. Then, the pure standard solution of the corresponding concentration was prepared and was detected directly, and the peak area of omeprazole was recorded as B. The absolute recovery was calculated by A/B ^*∗*^ 100%. The absolute recoveries of low, medium, and high concentrations of omeprazole were (81.01 ± 4.25) %, (83.17 ± 3.71) %, and (85.17 ± 3.22) %, respectively.

### 3.5. Sample Stability

The low, medium, and high concentrations (10, 250, 750 ng/ml) of omeprazole plasma standard solution were prepared with 6 copies of each concentration. The stability of plasma samples stored at room temperature and frozen storage, the stability of plasma samples under freeze-thaw conditions, and the stability of treated plasma samples in an automatic sampler were investigated. The results showed that omeprazole had good stability under four conditions, and the RSD was less than 10%.

### 3.6. The Pharmacokinetics of Omeprazole

**Figure 2:**
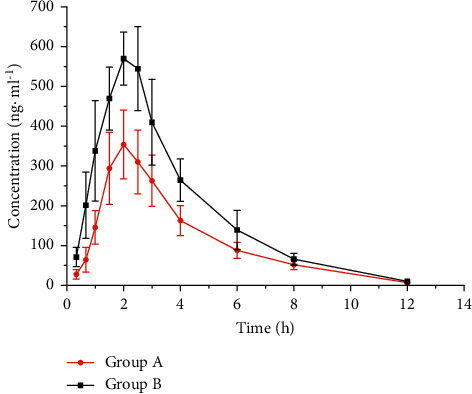
Plasma mean concentration-time curve of omeprazole in group A and B. Group A: omeprazole alone administration; group B: SJZPs combined with omeprazole.

The plasma drug concentration-time curve of omeprazole administration alone to six beagle dogs (group A) and SJZPs combined with omeprazole administration to six beagle dogs (group B) is shown in Figure 2. The plasma drug concentrations of omeprazole in group A and B were calculated by DAS 2.0. The main pharmacokinetic parameters of omeprazole are shown in Table 2.

### 3.7. The Effect of SJZPs on the Pharmacokinetics of Omeprazole

The *C*_max_ of omeprazole in group B was 61.55% higher than that in group A, and the AUC_(0−t)_ and AUC_(0−∞)_ of omeprazole in group B were 63.96% and 63.65% higher than those in group A, respectively. At the same time, CL and Vz decreased in group B. The results showed that SJZPs could slow down the metabolism of omeprazole and increase the plasma drug concentration of omeprazole in beagle dogs.

## 4. Discussion

Omeprazole is very unstable under acidic conditions and degrades quickly, and it is stable in methanol solution and under alkaline conditions, so the standard stock solution was prepared with methanol in the detection process. When preparing the application solution, omeprazole was diluted with sodium hydroxide solution. After the sample was extracted, it was redissolved with acetonitrile aqueous solution containing sodium hydroxide to ensure the stability of omeprazole in the resolution. In the detection process, the wavelength gradient of the DAD detector was adopted, and the detection wavelength was 265 nm in 1–6 min and 302 nm in 6–8 min, so as to ensure the maximum absorption of omeprazole and IS and avoid the interference of endogenous substances.

In this study, a HPLC-UV method for the determination of omeprazole concentration on plasma was established, and the method had high specificity, complete separation, and low cost. The intraday and interday precision were less than 7.46%, which met the basic requirements of the guiding principles of pharmacokinetics on biological sample analysis methods. The method could be used for the detection of drug concentration in the study of omeprazole pharmacokinetics and drug interaction.

Many herbs interact with drugs through a complex CYP450 and/or P-glycoprotein mechanism. Herb-induced enzyme inhibition and/or induction may result in enhanced and/or decreased plasma, tissue, urine, and bile drug concentrations, leading to a change in a drug's pharmacokinetic parameters and resulting in the improper treatment of patients and potentially severe side effects. Use of an appropriate method for comprehensively assessing HDIs can minimize clinical risks [22]. The majority of potential herb-drug interactions occur on a pharmacokinetic level [23]. For example, the herd Xiao-Ai-Ping Injection could inhibit the metabolism of enasidenib and increased the concentration of enasidenib in rats [24].

The results of this study showed that omeprazole was used in combination with SJZPs, the *C*_max_ of omeprazole in group B was 61.55% higher than that in group A, and the AUC_(0−t)_ and AUC_(0−∞)_ of omeprazole in group B were 63.96% and 63.65% higher than those in group A, respectively. At the same time, CL and Vz decreased in group B. The results showed that SJZPs could slow down the metabolism of omeprazole and increase the plasma drug concentration of omeprazole in beagle dogs. The concentration of omeprazole increased, and the curative effect was enhanced. This might be one of the effective mechanisms of combined application. However, the increase of concentration would also be accompanied by the increase of adverse reactions. Therefore, when SJZPs were combined wtih omeprazole clinically, the herb-drug interaction and adverse reactions should be paid attention to, and the dosage should be adjusted if necessary.

## 5. Conclusions

In this study, an HPLC method for the determination of plasma omeprazole concentration was established. SJZPs could inhibit the metabolism of omeprazole and increase the concentration of omeprazole in beagle dogs. It is suggested that when SJZPs were combined with omeprazole, attention should be paid to the herb-drug interactions and possible adverse reactions.

## Figures and Tables

**Table 1:** The precision and accuracy of omeprazole in beagle dog plasma (*n* = 6, mean ± SD).

Added (ng/mL)	Intraday			Interday		
	Found (ng/mL)	RSD (%)	RE (%)	Found (ng/mL)	RSD (%)	RE (%)
5	5.08 ± 0.36	6.99	1.63	4.96 ± 0.37	7.46	−0.83
10	9.92 ± 0.69	6.95	−0.82	10.12 ± 0.55	5.44	1.18
250	248.45 ± 13.44	5.41	−0.62	252.49 ± 11.51	4.56	1.00
750	758.32 ± 19.83	2.61	1.11	748.60 ± 19.85	2.65	−0.19

**Table 2:** The main pharmacokinetic parameters of omeprazole in beagle dogs. Group A: omeprazole alone administration; group B: SJZPs combined with omeprazole (*n* = 6, mean ± SD).

Parameters	Group A	Group B
*C*_max_ (ng/mL)	366.69 ± 83.92	592.41 ± 42.74^*∗∗*^
*T*_max_ (h)	1.88 ± 0.26	2.17 ± 0.41
*t*_1/2_ (h)	1.85 ± 0.31	1.89 ± 0.32
CL_*z*_/*F* (L/h/kg)	0.50 ± 0.11	0.30 ± 0.04^*∗*^
*V*_*z*_/*F* (L/h)	1.73 ± 0.35	0.83 ± 0.23^*∗*^
AUC_(0−*t*)_ (ng·h/mL)	1359.81 ± 240.22	2229.67 ± 299.72^*∗∗*^
AUC_(0−∞)_ (ng·h/mL)	1386.37 ± 249.04	2268.83 ± 291.43^*∗∗*^

Abbreviations: *t*_1/2_, half-life; *T*_max_, time of peak concentration. *C*_max_, peak concentration. CL_*z*_/*F*, clearance. *V*_*z*_/*F*, apparent volume of distribution. AUC_(0−*t*)_, area under the curve of 0-*t* time. AUC_(0−∞)_, area under the curve of 0-infinity time (compared with group A, ^*∗*^*P* < 0.05 and ^*∗∗*^*P* < 0.01).

## Data Availability

The data used to support the findings of this study are available from the corresponding author upon request.
